# Subject-Specific Deep Learning Model for Motor Imagery Direction Decoding

**DOI:** 10.3390/s26185951

**Published:** 2026-09-20

**Authors:** Praveen K. Parashiva, Sagila Gangadharan Kutteri, A. P. Vinod

**Affiliations:** Infocomm Technology, Singapore Institute of Technology, Singapore 828608, Singapore

**Keywords:** direction decoding, MI, unilateral MI, brain–computer interface, BCI, deep learning, EEGNet

## Abstract

Hemispheric strokes impair motor control in contralateral body parts, necessitating effective rehabilitation strategies. Motor imagery-based brain–computer interfaces (MI-BCIs) promote neuroplasticity, aiding the recovery of motor functions. While deep learning has shown promise in decoding MI actions for stroke rehabilitation, existing studies largely focus on bilateral MI actions and are limited to offline evaluations. Decoding directional information from unilateral MI, however, offers a more natural control interface with greater degrees of freedom but remains challenging due to spatially overlapping neural activity. This work proposes a novel deep learning framework for online decoding of binary directional MI signals from the dominant hand of 20 healthy subjects. The proposed method employs EEGNet-based convolutional filters to extract temporal and spatial features. The EEGNet model is enhanced by squeeze-and-excitation (SE) layers that rank the electrode importance and feature maps. A subject-independent model is initially trained using calibration data from multiple subjects and fine-tuned for subject-specific adaptation. The performance of the proposed method is evaluated using subject-specific online session data. The proposed method achieved an average right vs. left binary direction-decoding accuracy of 58.7±8% for unilateral MI tasks, outperforming the existing deep learning models. Additionally, the SE-layer ranking offers insights into electrode contribution, enabling potential subject-specific BCI optimization. The findings highlight the efficacy of the proposed method in advancing MI-BCI applications for a more natural and effective control of BCI systems.

## 1. Introduction

A brain–computer interface (BCI) system promotes neural plasticity and supports the re-learning of lost functions in stroke patients by providing visual, auditory, or haptic feedback based on decoded brain signals [1]. In electroencephalogram (EEG)-based BCI systems, the neural activity is recorded non-invasively and the underlying information is decoded using signal processing and machine learning techniques [2]. Motor imagery (MI)-based BCI focuses on decoding the neural information due to imagination of the motor movement actions. Bilateral MI tasks such as left- vs right-hand elicit neural signatures in the non-overlapping brain regions, resulting in spatially separated EEG signals, and state-of-the-art methods such as the Filter Bank Common Spatial Pattern (FBCSP) [3] achieve acceptable classification accuracy for MI decoding. The existing MI-BCI research predominantly decodes bilateral MI actions and assigns artificial control commands to the external device [4]. However, the natural control of the external device requires MI actions that are closely related to the user’s thoughts. For instance, the steering of robots towards right vs left direction using the movement imaginations of the arm towards right or left directions is more natural compared to movement imagination of right arm vs foot MI actions. Further, decoding of direction-related information from a unilateral MI action increases the degrees of freedom. However, decoding direction-related information from EEGs is challenging due to the spatial overlap of the neural activity in the motor cortex region of the brain [5]. In addition, the inherent limitation of the poor spatial resolution of EEGs, its susceptibility to noise, and the limited dataset add to the challenges for decoding direction-related information from MI tasks [2]. This work focuses on decoding direction-related information from EEGs during unilateral MI actions and evaluating the performance in an online experimental setup.

In the existing literature, the decoding of unilateral MI actions relies on feature engineering and machine learning methods. In [6,7,8], the EEG data from the unilateral motor execution tasks suggested that the direction-related information is captured using phase-related and variance features from the very-low-frequency (<10 Hz) and high-frequency (>60 Hz) signals. The direction information from unilateral motor imagination tasks is extracted using Phase-Locking Value (PLV) and Common Spatial Pattern (CSP) features from the wavelet levels corresponding to lower and higher frequency bands in [9,10]. The feature engineering approach relies heavily on the design of features tailored on a subject-specific dataset, including channel optimization [11], feature ranking [12], and classifier design [13]. However, these methods often necessitate extensive manual feature engineering and are less robust. Deep learning models have become increasingly popular because of their ability to generalize and perform end-to-end learning of latent information, and these approaches hold great promise for providing deeper insights into EEG data. However, deep learning models typically require large datasets to achieve optimal performance, whereas EEG datasets are often limited in size and prone to high levels of noise, posing significant challenges to their effective application.

In the existing literature, the number of layers and filters of the deep learning architecture used in EEG-based BCI applications is less than that in computer vision models such as ResNet and Inception [14]. EEG is a multi-channel time-series signal, and the deep learning model needs to learn the temporal and spatial information efficiently for BCI applications. Shallow and Deep ConvNet model architectures use convolution operation along the time and electrode axis of the EEG signal to extract temporal and spatial information in a bilateral MI task [15]. Deep ConvNet achieved comparable classification accuracy to that of FBCSP. EEGNet [16] used depth-wise and separable convolution operations to extract temporal and spatial information efficiently and showed robustness in BCI paradigms such as MI and P300. Inspired by FBCSP, an end-to-end model referred to as FBCNet pre-filters the EEG data into eight frequency bands, and a temporal log-variance layer is introduced to extract CSP-like features [17]. The FBMSNet model [18] uses filter bank, temporal convolution, spatial convolution, and temporal log-variance layers with a combination of central loss and cross-entropy loss for decoding bilateral MI actions. Many existing deep learning model studies on MI-BCI rely on openly available bilateral MI task datasets such as BCI Competition IVa to train a subject-independent offline model and report Leave One Out Validation (LOOV) accuracy [19,20,21]. Subject-specific models trained using feature engineering approaches have shown improved performance by ranking electrode importance and the extracted features using Fisher’s ratio, mutual information, correlation, etc. [11]. The existing deep learning methods for decoding MI do not consider the importance of electrodes and feature maps created using filters.

As the number of trials in MI-BCI experiments is less than 100 per subject, deep learning models are often trained on multiple subjects’ data to handle this data scarcity. Further, transfer learning methods are employed to fine-tune the model [22]. Riemannian Alignment (RA) and Euclidean Alignment (EA) attempt to adapt the covariance matrix of the target domain with that of the source domain in Riemannian and Euclidean space, respectively [23]. The common transfer learning approach employed in deep learning models is to fine-tune the pre-trained model on target domain data. Fine-tuning the weights of specific layers such as dense layers using target domain data is shown to achieve improved performance [19,21].

Based on a review of the existing deep learning literature on MI-BCI, the following observations can be made: (1) the current deep learning models have demonstrated promise in decoding EEG signals, particularly for bilateral MI tasks, (2) convolution-based architecture typically includes at least two layers to extract temporal and spatial information, and (3) these models are often trained on subject-independent datasets. There is a growing research interest in decoding unilateral MI tasks, such as directional information. Recent advancements include the use of extensively preprocessed and filter-banked EEG signals processed through self-attention layers applied to temporal and spatial convolution filters. This approach has achieved approximately 59% accuracy in decoding left- vs right directional MI tasks from the unilateral limb [24]. Further, the subject-independent model was trained using 46 subjects, leveraging a substantial data advantage to enhance model performance.

The research on improving MI-BCI classification accuracy has been predominantly supported by the public datasets focused on bilateral limb activities [25,26]. However, the overlapping brain regions involved in unilateral limb movements pose significant challenges, limiting the performance of models trained on bilateral MI tasks. To address these challenges, a more refined approach is essential. This work introduces a novel deep learning framework designed to enhance the decoding of unilateral MI tasks, emphasizing the assignment of importance to electrodes and feature maps derived from temporal and spatial convolution filters. The primary contributions of this work are:

1. Development of a novel deep learning model for binary directional decoding of unilateral MI tasks, evaluated on online session data.
2. Integration of squeeze-and-excitation (SE) layers [27] to rank the importance of EEG electrodes and feature maps generated by convolution filters.
3. Subject-specific models’ optimization through fine-tuning of SE layers, leading to improved performance.
4. Comprehensive evaluation of subject-specific and subject-independent models, using online session data and benchmarking against state-of-the-art methods.

The paper is structured as follows: Section 2 outlines the proposed methodology, detailing the experimental dataset and the novel model architecture. Section 3 presents the results achieved with the proposed method, including comparisons with existing approaches. Section 4 discusses the limitations, future directions, and concluding remarks.

## 2. Methodology

The section presents the details of the experimental setup and the dataset for the unilateral binary MI direction task followed by a novel deep learning architecture for direction decoding.

### 2.1. Experimental Setup and Dataset Details

**Figure 1:**
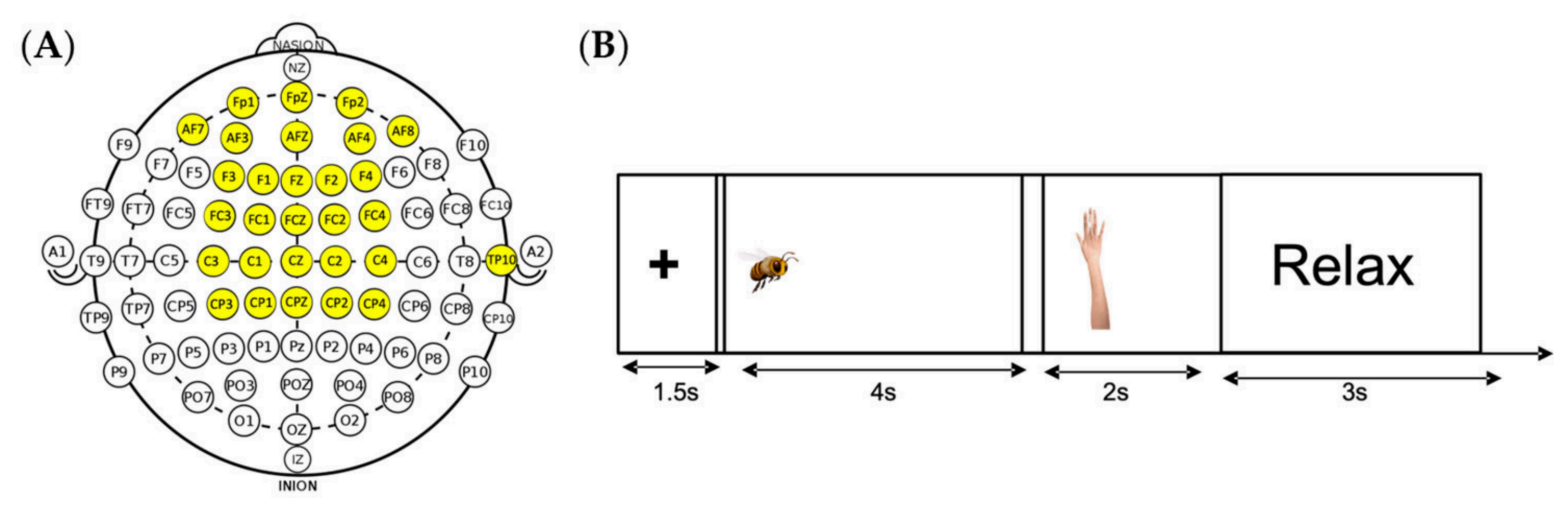
(**A**) Electrode placement and (**B**) timing diagram. The electrodes highlighted in yellow are the 27 electrodes considered for EEG recording.

Twenty healthy subjects (eleven male, nine female; age: 27.9 ± 2.5) participated, of which the dominant hand of two subjects (one male and one female) was the left hand and that of the remaining eighteen subjects was the right hand. During the experiment, subjects were seated on a comfortable cushioned chair with an armrest, facing a computer monitor and with their dominant hand placed on the computer table. The subjects were asked to perform the center-out motor imagination of their dominant hand, in either the left or right direction. While the subjects performed MI actions, their EEG data were recorded using 27 Ag/AgCl electrodes with respect to an electrode placed at the right ear mastoid, referred to as TP10. The electrode placement is shown in Figure 1A. Further, the data were recorded at a sampling frequency of 500 Hz using an actiCHamp amplifier ( BrainProducts GmbH, Gilching, Germany).

A visual cue was presented on the computer screen to guide the user to perform unilateral MI actions. The timing diagram for cue presentation is illustrated in Figure 1B. Each trial commenced with a fixation cue lasting 1.5 s, followed by a motor imagery cue that was displayed for 4 s. This was succeeded by a feedback cue for 2 s, after which participants were allowed to relax for 3 s. More details of the experimental setup can be found in [28]. The EEG data correspond to the 4 s MI cue and 2 s feedback carrying underlying brain-related information. In general, the MI action performed by the subject was decoded using the 4 s MI EEG data alone. In [28], the 2 s feedback data were also used to improve the direction-decoding accuracy. In this work, the EEG data corresponding to the MI cue alone were considered, ignoring the EEG data due to the visual feedback. The EEG data were recorded using Lab Streaming Layer (LSL) (v1.13).

The experiment comprised two sessions—calibration and online—both conducted on the same day with a brief interval of approximately 10 min. The calibration session was conducted first, involving all 20 subjects (S01 to S20), with each subject participating in 72 trials, consisting of 36 trials per class. Following the calibration, the online session was performed exclusively with subjects S08 to S20, comprising 48 trials, split evenly with 24 trials per class. During the calibration session, the feedback cue was pre-programmed to simulate a direction decoding model with an accuracy of 75%. This is based on an assumption that the existing MI decoding model achieves a classification accuracy of ~75% [29]. In contrast, the feedback during the online session was derived from a model trained on the specific calibration data of each subject [28]. It is important to note that feedback data from both the calibration and online sessions were not utilized in this work. Representative single-trial EEG traces (all 27 channels, with C3 and C4 highlighted) and their frequency-domain power spectral density analysis for this dataset are provided in the project repository (https://github.com/Parshiva24/EEGNetSE) (accessed on 14 September 2026).

### 2.2. Proposed Model Architecture

**Figure 2:**
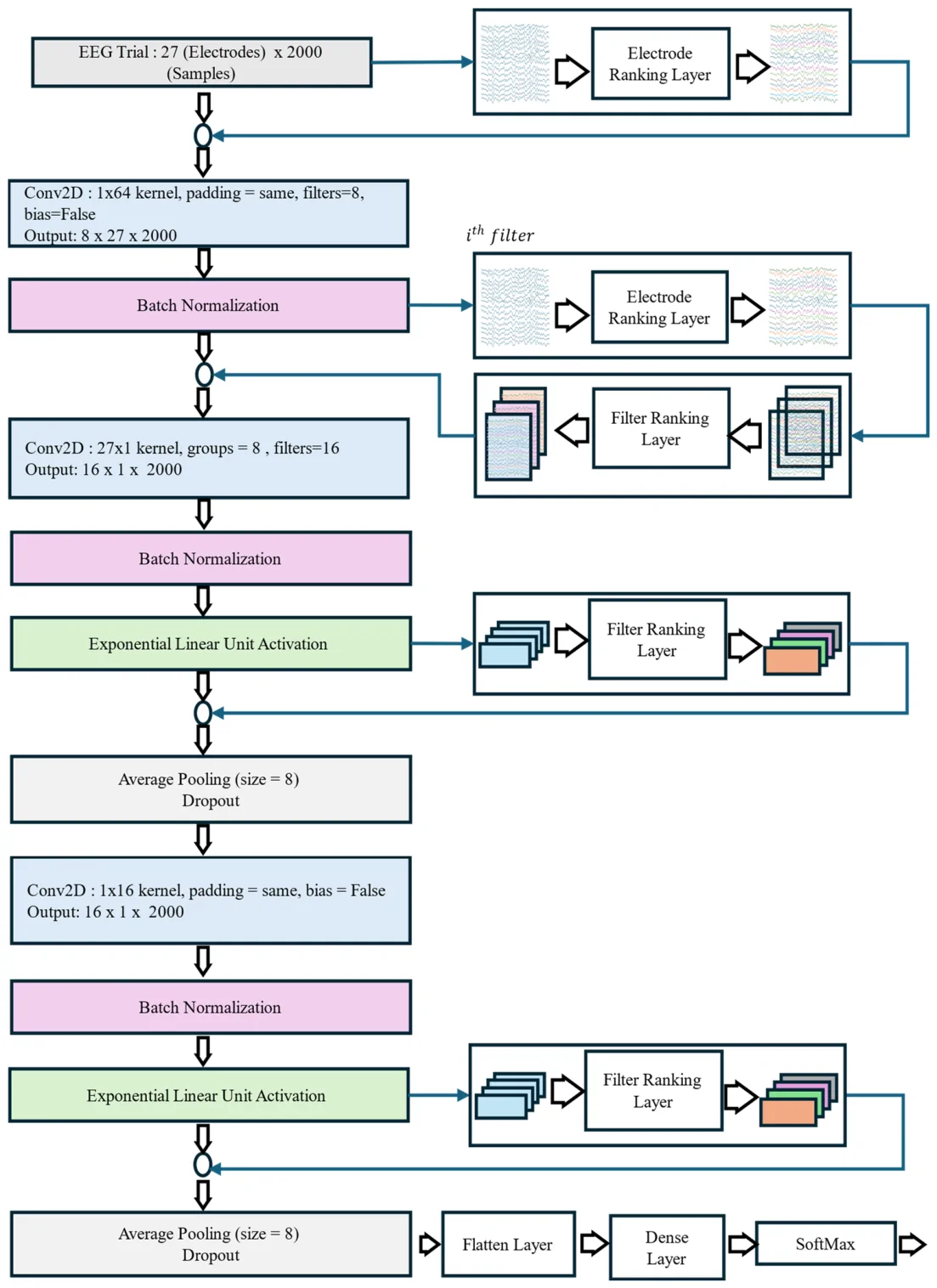
Block diagram of the proposed model. The input to the Electrode and Filter ranking layer is shown with same color whereas, the outputs are shown in different colors to represent the ranking.

The EEG data were pre-filtered using a 5th-order Butterworth bandpass filter between 0.5 Hz and 90 Hz followed by an IIR notch filter to remove 50 Hz line noise. The signal was then baseline corrected and re-referenced using Common Average Referencing (CAR). The preprocessed EEG signal was then input into the proposed model, as illustrated in Figure 2. The proposed model incorporates convolution filters, electrode and feature map ranking layers using SE block, along with pooling, batch normalization and activation layers. The convolution filters are based on the EEGNet architecture, depth-wise convolution to extract temporal features, and spatial convolution to capture spatial information. Non-linearity is introduced via an Exponential Linear Unit (ELU) activation function, while regularization is achieved using an average pooling layer followed by a dropout layer.

The SE block is utilized to implement the electrode and feature map ranking layers in a fully end-to-end learnable deep learning model. In the electrode ranking layer block depicted in Figure 2, the input EEG electrode data are visually represented using uniform-colored blue lines, signifying equal weights for all electrodes. After processing through the SE block for an electrode, the output data are represented with varying colors reflecting the ranking assigned to each electrode. Similarly, in the feature map ranking layer, input and output are color coded to illustrate the ranking assigned to each convolutional filter. This ranking mechanism enhances the model’s ability to focus on the most relevant electrodes and feature maps, thereby improving the overall decoding process. The ranked features are further processed through convolutional layers to extract spatiotemporal patterns followed by batch normalization, activation, and pooling layers, as shown in Figure 2. The resulting features are flattened and passed through a dense layer with a SoftMax activation function applied to classify each EEG trial as either left- or right-direction. The proposed architecture integrates ranking and feature extraction seamlessly, enabling more accurate and interpretable classification of EEG data.

### 2.3. Electrode Ranking Layer

**Figure 3:**
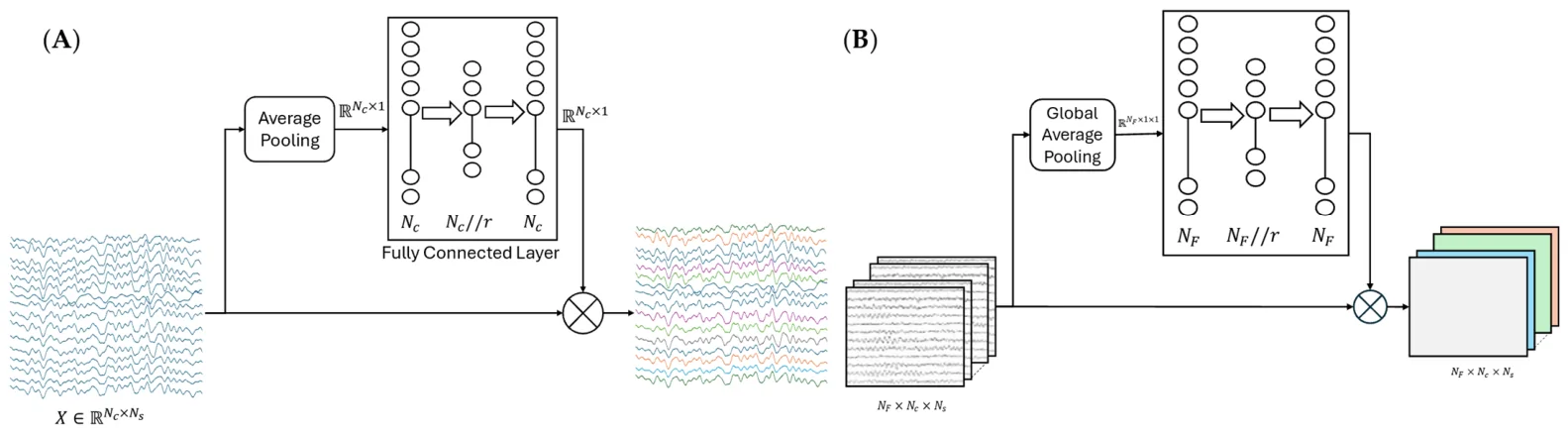
(**A**) Electrode ranking layer and (**B**) feature map ranking layer.

In computer vision, the SE block is traditionally used to scale the convolution-filtered signals to enhance model performance [27]. This work extends the SE block to rank EEG electrodes based on their importance, thereby improving the performance of MI-BCI models. The proposed electrode ranking layer, illustrated in Figure 3A, operates as follows: Given a 2D EEG signal, $X\in R^{N_{c}\times N_{s}}$, where $N_{c}$ and $N_{s}$ represents the number of electrodes and samples, the electrodes are respectively ranked through average pooling and a fully connected layer with a sigmoid activation at the output. The average pooled value $z_{c_{i}}$ for the $i^{th}$ electrode is computed as the mean of all sample values in that electrode, as given in Equation (1). The resulting pooled vector is $z_{c}\in R^{N_{c}\times 1}$. The pooled vector $z_{c}$ is passed through a dense network with one hidden layer and an output layer. The hidden layer has $N_{c}//\;r$ units where r is the reduction rate, and the output layer has $N_{c}$ units. The activation functions for the hidden and output layers are ReLU and sigmoid, respectively. Let $W_{1}\in R^{\frac{N_{c}}{r}\times N_{c}}$ and $W_{2}\in \;R^{N_{c}\times \frac{N_{c}}{r}}$ represent the weights of the hidden and output layers. The output $s_{c}\in R^{N_{c}\times 1}$ representing the ranking or scaling factors for the electrodes is computed in Equation (2). The input EEG signal X is scaled using the computed ranks $s_{c}$, producing the scaled output $X_{scaled}$ as in Equation (3).

(1) $$z_{c_{i}}=\frac{1}{N_{s}}\;\sum_{j=1}^{N_{s}}X\left(c_{i},\;j\right)$$

(2) $$s_{c}=sigmoid(W_{2}\times ReLU\left(W_{1}\times z_{c}\right))$$

(3) $$X_{scaled}=s_{c}⨀X$$

In the proposed model (Figure 2), the electrode ranking layer is applied to the preprocessed EEG signal, $X\in R^{N_{c}\times N_{s}}$ and to the output signal of the depth-wise convolution filter, $Y\in R^{{N_{F}\times N}_{c}\times N_{s}}$, where $N_{F}$ is the number of filters. Since the electrode ranking layer operates on 2D signals, it processes each feature map in Y individually. For each feature map $Y_{f}\in R^{N_{c}\times N_{s}}$ (where f indexes the feature maps, $f=1,\;2,\;\ldots N_{F})$, the electrodes are ranked using the same methodology described for X, thus enhancing the ability of the model to capture spatially significant patterns at multiple stages of feature extraction.

### 2.4. Feature Map Ranking Layer

In existing models such as EEGNet, Deep ConvNet, FBCNet, and FBMSNet, feature maps represent spatial, temporal, or hybrid information extracted using various filters, with the number of filters $N_{F}$ treated as a hyperparameter. However, these models do not prioritize or rank the feature maps produced by these filters, even when trained end-to-end. The SE block for feature map ranking is like the electrode ranking layer described in Section 2.3. The feature map ranking layer, shown in Figure 3B, operates as follows:

Let the output of the convolutional layer be $Y\in R^{{N_{F}\times N}_{c}\times N_{s}}$. The ranking process begins by global pooling Y across its spatial and temporal dimensions $(N_{c}$ and $N_{s})$ to produce a vector $z_{F}\in R^{N_{F}\times 1}$, computed as in Equation (4). The pooled vector $z_{F}$ is then passed through a dense network consisting of a hidden layer and an output layer. The hidden layer contains $N_{F}//r$ neurons (where r is the reduction rate), and the output layer contains $N_{F}$ neurons. The activation functions for the hidden and output layers are ReLU and sigmoid, respectively. The feature map ranks $s_{F}\in R^{N_{F}\times 1}$ are calculated as Equation (5). Finally, the original feature maps Y are scaled by the computed ranks $s_{F}$, resulting in the scaled output $Y_{scaled}$ as in Equation (6), where ⨀ denotes element-wise multiplication.

(4) $$z_{F}=\frac{1}{N_{c}\times N_{s}}\;\sum_{i=1}^{N_{c}}\sum_{j=1}^{N_{s}}X(i,\;j)$$

(5) $$s_{F}=sigmoid(W_{2}\times ReLU\left(W_{1}\times z_{F}\right))$$

(6) $$Y_{scaled}=s_{F}⨀Y$$

By ranking the feature maps, the proposed approach allows the model to prioritize the most informative filters, improving its ability to capture relevant spatiotemporal patterns and enhancing overall performance in EEG decoding.

### 2.5. Training and Evaluation Protocol

The dataset is partitioned into training, validation, and test sets. The subject-independent base model is fitted once on the calibration trials of subjects S01–S07 using a 90/10 class-stratified split for training and early-stopping validation. For each target subject (S08–S20), 90% of the 72 calibration trials are used to fine-tune the base model and the remaining 10% for early-stopping validation. The 48 trials of the target subject’s online session form the test partition and are never seen during training or validation in either stage. The online session is recorded under a closed-loop condition that is physically and temporally separate from the open-loop, pre-programmed-feedback calibration session; consequently, all accuracies reported in Table 1 and thereafter are held-out generalization estimates against a separate recording rather than within-session cross-validation figures. Because a single base model is shared across all target subjects and no hyperparameter is selected using online session data, this protocol provides a stricter generalization test than k-fold cross-validation on the calibration data.

## 3. Results

The proposed deep learning model (Figure 2) is trained in two stages: subject-independent pretraining followed by subject specific fine-tuning. The base model (pre-training) used pooled calibration session data from S01–S07 using the Adam optimizer with a learning rate of $1\times 10^{-4}$, batch size of 32, and the categorical cross-entropy loss function with early stopping to prevent overfitting. The early stopping criteria include a patience of 30 epochs, a minimum delta (δ) of $1\times 10^{-3}$, and a minimum training epoch requirement of 100; the total epoch set is 1000. The fine-tuning of the base model is on subject-specific calibration data with the learning rate reduced to $1\;\times \;10^{-5}$, batch size of 16, and up to 200 epochs (early stopping patience 20). The convolutional filters remain frozen and act as feature extractors, and the SE and dense layers are updated. The reduction rate for the electrode and feature map ranking layers is set to 3, ensuring efficient feature selection during training. The experiments were performed using an NVIDIA RTX 2000 Ada GPU with CUDA 9.2, implemented in the PyTorch framework (v2.14).

The from-scratch and two-stage training strategies share the same optimizer, learning rate, batch size, loss, early stopping rule, validation-split scheme, and random seed. All the hyperparameters and regularizers remain the same for both from-scratch and two-stage training pipelines. The batch normalization (BN) statistics are computed from the training dataset; in the from-scratch mode the mean/variance parameters are learned from all the 72 calibration trials, whereas for the two-stage approach the BN statistics are inherited from the pre-training stage together with frozen convolutional kernels. As a standard fine-tuning process, the mean and variance of the BN statistics were re-estimated on the fine-tuning input trials.

### 3.1. Performance Analysis

The proposed deep learning model was trained on the unilateral MI direction dataset described in Section 2.1. This section presents the decoding accuracy of unilateral MI directions using two types of model: a subject-independent model and a subject-specific model. The results of both models, along with comparisons to existing methods, are reported.

**Figure 4:**
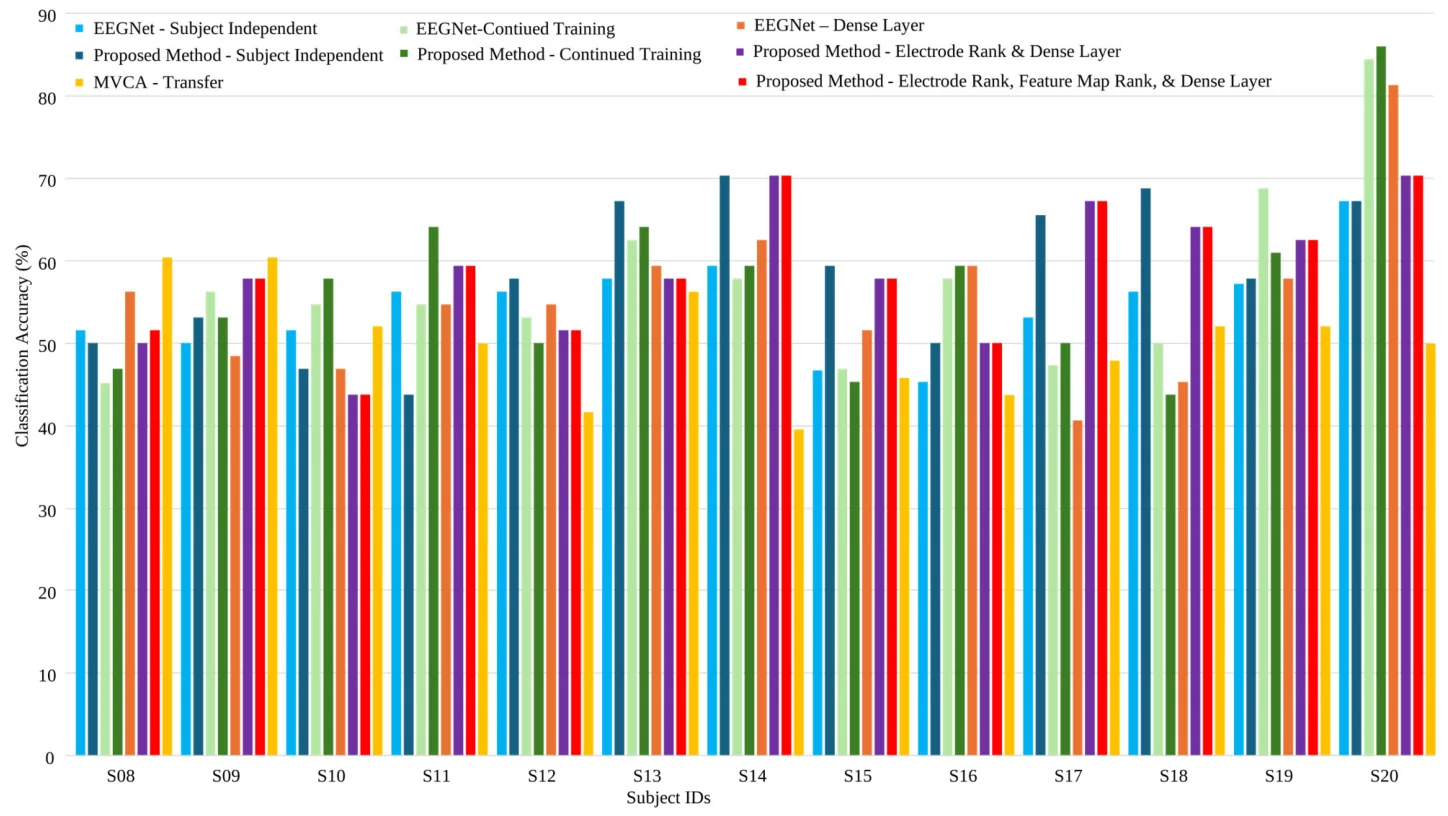
Comparison of online unilateral MI direction-decoding accuracy.

The subject-independent model, referred to as the base model, was trained using calibration session data from subjects S01 to S07, who did not participate in online sessions. Subjects S08 to S20 participated in both calibration and online sessions. The base model is used to infer the unilateral MI direction-decoding accuracy for the online session data of subjects S08 to S20. The average decoding accuracy achieved by the base model is presented in Table 2, which also includes the comparison to the accuracies achieved by existing models. Figure 4 illustrates the online MI direction-decoding accuracy for subjects S08 to S20, with results from EEGNet and the proposed method depicted in light- and dark-blue bars, respectively. The average online MI direction-decoding accuracy using the proposed subject-independent model is 58.28±9.00%, outperforming existing state-of-the-art deep learning methods such as EEGNet [16] and FBCNet [17]. Additionally, Table 2 also compares the results of the base model to those obtained using the Wavelet CSP (W-CSP) method [9] and the combination of W-CSP and W-PLV features [28], which rely on subject-specific parameter tuning. The proposed subject-independent base model achieves higher direction-decoding accuracy compared to the existing feature engineering approach.

To benchmark the proposed method against a recent state-of-the-art model, the multi-view convolutional self-attention (MVCA) network [24], originally proposed for four-class unilateral-limb MI direction decoding, was re-implemented on the present dataset. Following the reported architecture, MVCA was re-implemented using a nine-band filter bank (4–40 Hz in 4-Hz bands), per-band temporal and spatial convolutions, multi-head self-attention with projections shared across bands, and a linear SoftMax classifier (~700,000 parameters, compared with 8432 for the proposed model). MVCA was evaluated the same two-stage pre-training/fine-tuning protocol used for the proposed model—on each subject’s held-out online session. As reported in Table 2, the re-implemented MVCA achieved 50.16 ± 6.25% (two-stage), lower compared to the proposed method. In [24], the MVCA method is reported to achieve a classification accuracy of 59.86% for the closest comparable binary (1:2 pair) MI tasks. The difference in classification accuracy is largely attributed to the data scale. Other recent deep models, such as attention temporal convolutional networks [30], squeeze-and-excitation multi-branch CNNs [31], and weight-optimized models for unilateral upper-limb MI [32], similarly depend on substantially larger datasets, whereas transferring a pre-trained model across subjects has proven effective when subject-specific data are scarce [33]. Further, the performance also depends on the subjects’ capability to modulate the neural signals [34,35]. MVCA is a large model and is re-implemented matching the original architecture and the hyperparameters (*m* = *n* = 32, *w* = 64, *h* = 2, *head-dim* = 8) reported in [24], but trained on a ~30-times-smaller dataset compared to [23]; therefore, the drop in classification accuracy for the re-implemented MVCA method is expected. Further, the dataset used in [24] is publicly not available currently; therefore, MVCA and EEGNetSE are trained on the same dataset and compared in Table 2. In future, the performance of the proposed EEGNetSE will be evaluated on the 46-subject dataset should the dataset become available.

The subject-specific model for unilateral MI direction decoding is obtained by fine-tuning the base model. The base model, trained on the calibration session data of subjects S01 to S07, serves as a feature extractor. Subject-specific models are created by updating the weights of selected layers in the base model using subject-specific calibration data. In this work, the convolution filter weights of the base model remain fixed, acting solely as feature extractors, while the weights of the proposed electrode ranking and feature map ranking layers are fine-tuned using the subject-specific calibration dataset. For comparison, the dense layer of EEGNet is fine-tuned using the same subject-specific calibration data. The direction-decoding accuracy achieved using subject-independent and subject-specific models achieved with fine-tuned models is presented in Figure 4. The light- and dark-blue bars show the subject-independent direction-decoding accuracies achieved using EEGNet and the proposed method, respectively. The light- and dark-green bars represent the performance of the subject-specific calibration data with EEGNet and the proposed method, respectively. Additionally, the fine-tuned direction-decoding accuracies achieved by updating the dense layer of EEGNet, the dense and electrode ranking layers of the proposed model, and the feature map ranking layers of the proposed model are shown as orange, purple, and red bars, respectively. The average MI direction-decoding accuracies of the subject-specific models are summarized in Table 1.

Table 1 compares five fine-tuning configurations that differ in which layers are updated on the target subject; the added column lists the layers updated in each case. In “EEGNet–Continued Training”, all layers of the pre-trained EEGNet base model are unfrozen and retrained on the target subject’s calibration trials, whereas in “EEGNet–Dense Layer”, the convolutional and batch-normalization layers are frozen and only the final dense layer is updated. For the proposed model, “Continued Training” unfreezes all layers (convolution, SE, and dense); “Electrode Ranking and Dense Layer” freezes the convolutional filters and updates only the SE electrode-ranking and dense layers; and “Electrode Ranking, Feature Map Ranking and Dense Layer” additionally updates the SE feature map ranking layer, which is the configuration recommended in this work. All five configurations use the fine-tuning hyperparameters described above.

The subject-specific model created by fine-tuning the proposed electrode ranking and feature map ranking layers achieved comparable or superior average online MI direction-decoding accuracy, as shown in Table 1. While the subject-specific EEGNet model, fine-tuned by updating the dense layer, outperformed its subject-independent counterparts, its performance was inferior to the proposed subject-specific models. The proposed method demonstrated equal or improved performance in 8 out of 13 subjects compared to the subject-independent model, with the highest improvement of 15.63% observed for S11. The overall improvement achieved by the subject-specific model compared to the subject-independent model was 0.5±0.9%. These results underscore the advantage of fine-tuning the proposed electrode and feature map ranking layers for enhancing subject-specific decoding performance.

To quantify the benefit of the two-stage approach, an ablation was performed in which a subject-specific model was trained from scratch for each target subject (S08–S20) using only that subject’s 72 calibration trials, with all layers randomly initialized and trainable, and with the architecture, preprocessing, and optimization settings identical to the proposed model. Evaluated on the same held-out online sessions, training from scratch achieved an average accuracy of 54.17%, approximately 4.5% lower than the 58.65% obtained by the proposed two-stage (pre-train and fine-tune) model. This confirms that cross-subject pre-training provides a better-regularized starting point than subject-specific training from scratch, whose full-model optimization over-fits the limited per-subject calibration data.

### 3.2. Analysis of Electrode Ranking Layer

**Figure 5:**
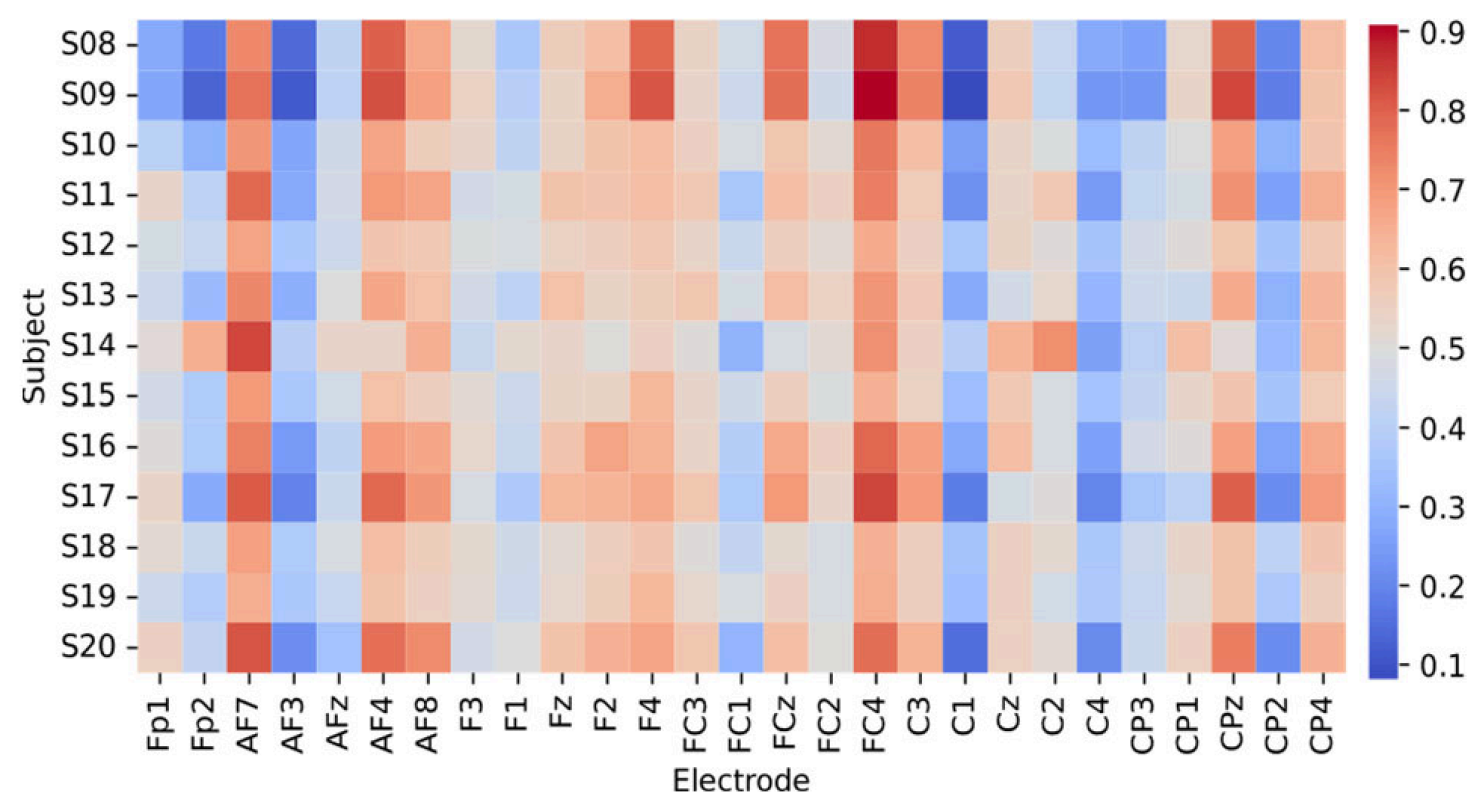
Heatmap to illustrate the electrode ranking layer on subjects S08 to S20.

The proposed electrode ranking layer was applied to the 2D EEG signal at the initial stage of the model, as shown in Figure 2. The results and analysis in this section are limited to the ranks assigned by the electrode ranking layer at the initial stage of the proposed model (Figure 2). To evaluate the rank assigned to each EEG electrode, the online session data of S08 to S20 were processed through the proposed model. The rank assigned to the electrodes, calculated using Equation (1), are visualized as heatmaps for each subject in Figure 5. The assigned ranks are color coded, with shades of blue indicating lower ranks and shades of orange indicating higher ranks; for instance, the heatmap reveals that the proposed method consistently assigns a lower rank to electrode C1 compared to C3 across all subjects.

The heatmap analysis further reveals a consistent pattern in ranking of electrodes across subjects. Electrodes such as Fp1, Fp2, AF3, AFz, C1, C4, and CP2 are consistently assigned lower ranks, whereas electrodes proximal to the motor cortex, including FC3, FCz, FC4, C3, Cz, C2, CP1, CPz, and CP4 are assigned higher ranks (Figure 5). These findings align with the motor-related cortical activity typically associated with MI tasks. Higher ranks assigned to electrodes such as AF7 are attributed to noise, as the proposed work did not deeply investigate the preprocessing of the signals of eye-related artifacts.

Performance analysis shows the subject-specific models obtained by fine-tuning the electrode and feature map ranking layers achieve the highest average direction-decoding accuracy (Table 1). The higher decoding accuracy of 70.31% is achieved for subjects S14 and S20, whereas the lowest accuracy of 43.75% is achieved for subject S10. Visual inspection of the heatmaps for subjects S14 and S20 indicates that electrodes FC1 and FCz are assigned notably lower ranks, while electrode C2 is assigned a higher rank compared to other subjects. Conversely, in the case of S10, FC1 is assigned a higher rank, while CP1 receives a lower rank compared to S14 and S20. These observations suggest that the ranks assigned to electrodes play a crucial role in influencing the direction-decoding accuracy of MI tasks. The ability of the model to fine-tune electrode importance in a subject-specific manner likely contributes to improved decoding performance by tailoring the model to individual neural activation patterns.

### 3.3. Analysis of Feature Map Ranking Layer

**Figure 6:**
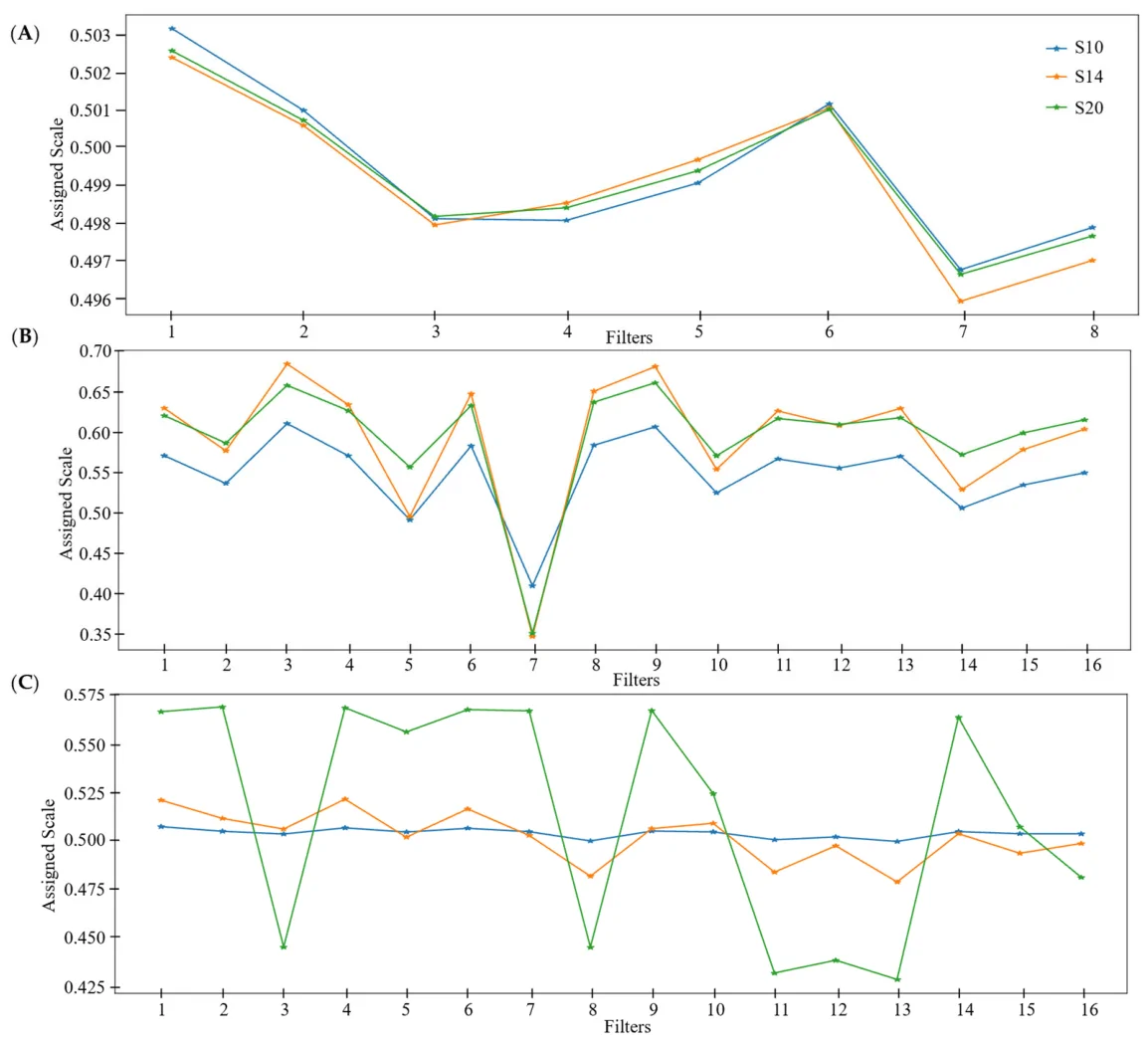
Filter rank assigned at (**A**) Layer 1, (**B**) Layer 2, and (**C**) Layer 3 in the proposed model.

The proposed model employs three sequential convolution layers with 8, 16, and 16 filters, respectively. The filtered signals generated by these convolution layers are scaled using the values computed by the feature map ranking layer. To analyze the ranks assigned to each filter, the output of the sigmoid activation function in the feature map ranking layer ($s_{F}$, as defined in Equation (5)) is plotted for subjects S10, S14, and S20 in Figure 6. The subjects were chosen because S14 and S20 achieved the highest direction-decoding accuracy, while S10 recorded the lowest accuracy using the proposed method. In Figure 6, the blue, orange, and green line plots represent the ranks assigned to the convolution filters for S10, S14, and S20, respectively, with the subplots corresponding to the filters in the first, second, and third convolution layers.

The analysis reveals that the ranks assigned to all the filters in the first convolution layer are nearly identical across all three subjects. However, significant differences are observed in the ranks assigned to the filters in the second convolution layer between high-performing (S14 and S20) and low-performing (S10) subjects. For instance, filter 7 in the second convolution layer is assigned a lower rank with a value of 0.35 for S14 and S20, whereas it is assigned a higher value of 0.40 for S10. Moreover, the average scale value (s_F) for S14 and S20 in the second convolution layer is approximately 0.60, compared to around 0.53 for S10, suggesting that higher-performing subjects emphasize different filter contributions compared to low-performing ones.

In the third convolution layer, the ranking patterns for the filters also differ significantly. For S10, all filters are assigned nearly equal ranks, whereas S14 and S20 display varying ranks among the filters in this layer. This indicates that the feature map ranking layer in the proposed model enables the identification of subject-specific importance of filters, particularly in the second and third convolution layers. These findings suggest that the variability in ranking among high-performing subjects could reflect their distinct neural activation patterns, emphasizing the importance of feature map scaling for improving MI task decoding.

### 3.4. Computational Complexity and Model Size

The computational cost of the proposed model was measured directly from the released code base (https://github.com/Parshiva24/EEGNetSE) (accessed on 14 September 2026). The model is lightweight, with 8432 trainable parameters (approximately 33 KB in single precision) and 61.1 MFLOPs (30.57 million multiply–accumulate operations) per trial. Of the 8432 parameters, 3312 (39.3%) are updated during subject-specific fine-tuning, while the convolutional backbone remains fixed. The measured single-trial inference time is 2.98 ms on an NVIDIA RTX 2000 Ada GPU and 26.1 ms on CPU, confirming that the model is suitable for real-time online BCI use. These figures are summarized in Table 3.

## 4. Conclusions

This work introduced a novel deep learning framework for decoding unilateral MI directions from EEG signals, addressing challenges posed by overlapping brain region activity and limited subject-specific model performance. By incorporating squeeze-and-excitation (SE) blocks for ranking EEG electrodes and feature maps in an end-to-end learning framework, the proposed method assigns importance to spatial and temporal features extracted by convolution filters, thereby improving classification accuracy. The electrode ranking layer identifies critical electrodes near motor cortex regions, while the feature map ranking layer emphasizes filters contributing most to task-specific decoding.

The experimental evaluation demonstrated that the proposed method achieved higher average decoding accuracy compared to state-of-the-art models such as EEGNet and FBCNet. The subject-independent model, trained using calibration data from multiple participants, outperformed traditional handcrafted methods like W-CSP and W-PLV. Furthermore, the subject-specific model, obtained by fine-tuning the electrode and feature map ranking layers, showed improved performance for the majority of subjects, achieving the highest direction-decoding accuracy of 70.31% and a significant improvement in certain subjects. The analysis of the ranking layers revealed that the model adapts dynamically to subject-specific neural patterns, underscoring the potential of the proposed method to capture individual differences in neural activity.

The proposed method has certain limitations. First, the decoding accuracy for some subjects remains low, suggesting the need for further optimization in handling individual variability. Second, while the SE-based ranking layers enhance model interpretability, an additional computational overhead is introduced by these layers. Additionally, the subject-specific model relies on calibration data for fine-tuning, which contains fewer trials and may not be feasible in real-world scenarios. Future work will focus on optimizing the ranking mechanism, exploring transfer learning to reduce dependency on extensive calibration data, and evaluating the model’s performance across larger and more diverse datasets. Furthermore, incorporating advanced preprocessing techniques to mitigate noise and artifacts in EEG signals could further enhance decoding performance.

## Figures and Tables

**Table 1:** Comparison of the performance of the fine-tuned subject-specific model.

Method	Classification Accuracy
EEGNet–Continued Training	56.87±10.53%
EEGNet–Dense Layer	55.28±10.05%
Proposed Method–Continued Training	56.97±11.15%
Proposed Method–Electrode Ranking and Dense Layer	58.65± 8.23 %
Proposed Method–Electrode Ranking, Feature Map Ranking and Dense Layer	58.77±8.10%

**Table 2:** Comparison of subject-independent online direction-decoding accuracy.

Method	Classification Accuracy
W-CSP [9] *	55.62±3.41%
W-CSP and W-PLV [28] *	54.91±3.81%
EEGNet [16]	54.50±5.75%
FBCNet [17]	49.99±6.73%
MVCA–two-stage transfer [24]	50.16 ± 6.25%
Proposed Method	58.28±9.00%

* Subject-dependent method.

**Table 3:** Computational complexity and model size of the proposed model.

Quantity	Value
Total parameters	8432
Trainable parameters (fine-tuning)	3312 (39.3%)
Multiply–accumulate operations per trial	61.1 MFLOPs
Inference time	2.98 ms (GPU), 26.1 ms (CPU)

## Data Availability

The raw EEG dataset used in this work is not public available due to participants privacy protocols but processed EEG trials and available from the corresponding author upon reasonable request.
